# Gli1 Is an Inducing Factor in Generating Floor Plate Progenitor Cells from Human Embryonic Stem Cells

**DOI:** 10.1002/stem.510

**Published:** 2010-08-26

**Authors:** Mark Denham, Lachlan H Thompson, Jessie Leung, Alice Pébay, Anders Björklund, Mirella Dottori

**Affiliations:** a Centre for Neuroscience, University of MelbourneParkville, Australia; b Department of Experimental Medical Science, Wallenberg Neuroscience Center, Lund UniversityLund, Sweden; c Florey Neuroscience InstituteParkville, Australia; d O'Brien InstituteParkville, Australia; e Department of Pharmacology, University of MelbourneParkville, Australia

**Keywords:** GLI1, Human embryonic stem cell, Parkinson's disease, Dopamine neuron

## Abstract

Generation of mesencephalic dopamine (mesDA) neurons from human embryonic stem cells (hESCs) requires several stages of signaling from various extrinsic and intrinsic factors. To date, most methods incorporate exogenous treatment of Sonic hedgehog (SHH) to derive mesDA neurons. However, we and others have shown that this approach is inefficient for generating FOXA2+ cells, the precursors of mesDA neurons. As mesDA neurons are derived from the ventral floor plate (FP) regions of the embryonic neural tube, we sought to develop a system to derive FP cells from hESC. We show that forced expression of the transcription factor GLI1 in hESC at the earliest stage of neural induction, resulted in their commitment to FP lineage. The GLI1+ cells coexpressed FP markers, FOXA2 and Corin, and displayed exocrine SHH activity by ventrally patterning the surrounding neural progenitors. This system results in 63% FOXA2+ cells at the neural progenitor stage of hESC differentiation. The GLI1-transduced cells were also able to differentiate to neurons expressing tyrosine hydroxylase. This study demonstrates that GLI1 is a determinant of FP specification in hESC and describes a highly robust and efficient in vitro model system that mimics the ventral neural tube organizer. Stem Cells 2010;28:1805–1815

## INTRODUCTION

In the last decade, since the derivation of human embryonic stem cell (hESC) lines, there has been much effort in generating mesencephalic dopamine (mesDA) neurons from hESC for developing cell replacement therapies to treat Parkinson's disease [1–3]. The ideal approach for deriving mesDA neurons from hESC is to mimic embryonic ventral midbrain development in culture. The ventralization aspect of the differentiation protocol is especially important as dopaminergic neurons are found within multiple regions in the brain and specific ventral markers need to be used to identify the “true” mesDA neuron. During mouse embryogenesis, the first postmitotic mesDA neurons can be identified by Nurr1 expression at E10.5 [4] and then later, at E11.5, are immunoreactive for tyrosine hydroxylase (TH) [5]. These TH neurons arise from the ventral regions of the mesencephalon [6,7], as identified by the expression of transcription factors known to be critical for the development of mesDA progenitor cells. FoxA2 is one such gene expressed in the mesDA progenitor cells and its expression is maintained in adult mesDA neurons [8,9]. Heterozygous *FoxA2* mutants show a progressive loss of mesDA neurons in adult mice indicating its requirement for mesDA survival [8].

In addition to mesDA progenitors, FOXA2 is expressed within the neighboring floor plate (FP) regions of the ventral neural tube [10]. The development of the FP is dependent on Sonic hedgehog (SHH) signaling. SHH is initially produced in the notochord during gastrulation, which in turn induces the neighboring neural tube FP to also secrete SHH, thereby creating a ventralizing signaling organizer [11]. In mice, loss of SHH reveal that it is a key morphogen required for the development of the FP [12] and its concentration gradient is instrumental in specifying the dorsoventral identity of cells throughout the neural tube [13]. Within the FP regions, where SHH concentrations are endogenously the highest, SHH induces expression of FoxA2 [14]. FoxA2 is a downstream target of Gli1 as previous studies in mouse embryos have shown that forced Gli1 expression in dorsal neural progenitors resulted in ectopic FoxA2 expression [15].

Obtaining a ventral identity of FP cells is a key step in the specification of midbrain neural cells toward a mesDA neural fate. Over years, there have been several protocols developed for inducing midbrain dopaminergic neurons from mouse embryonic stem (ES) cells and hESC [16–19]. Some of these methods involve supplementation of exogenous SHH or rely on endogenous SHH production and as well as other ventralizing factors within the cultures, to induce ventralization of neural progenitors [16–23]. The effectiveness of exogenous SHH treatment in ventralizing neural progenitors depends on the potency. Our data shows that exogenous treatment of C24II human recombinant Shh is ineffective in upregulating FOXA2 expression despite using a range of SHH concentrations and timing of treatment. Likewise, previous studies reported relatively low levels of FoxA2+ cells generated from SHH-treated neural progenitors derived from primate ES cells [21]. A more recent study from Fasano et al. [24] showed mouse recombinant Shh (C25II) to be significantly more effective in ventralizing human neural progenitors and generated FP cells. In our study, we undertook an intrinsic approach to upregulate FOXA2 expression and induce FP cells from hESC. Our studies show that inducing GLI1 expression in hESC, at the earliest stage of neurogenesis, results in their commitment to FOXA2+ FP progenitor cells at high efficiency. The hESC-derived FP cells were capable of patterning neighboring cells into a range of ventral cell types through the secretion of SHH. Furthermore, a proportion of these FP progenitor cells can further differentiate into ventral dopamine neurons. These studies were performed in a feeder-free culture system and demonstrate that GLI1 is a potent inducer of FP cells at the earliest onset of hESC neural differentiation. Overall, we describe the creation of an *in vitro* model system that mimics the ventral neural tube organizer.

## MATERIALS AND METHODS

### hESC Culture

HES-3 (Madison, WI, http://www.wicell.org), ENVY-HES-3 (Genome, Singapore, http://www.biotimeinc.com/esi/), and HUES-10 [25] cell lines were cultured as previously described [26,27]. Briefly, hESCs were culture on mitomycin-C treated mouse embryonic fibroblasts (MEFs) in hESC medium consisting of high-glucose Dulbecco's Modified Eagle Medium (DMEM) without sodium pyruvate, supplemented with insulin/transferrin/selenium 1%, β-mercaptoethanol 0.1 mM, nonessential amino acids (NEAA) 1%, glutamine 2 mM, penicillin 25 U/ml, streptomycin 25 μg/ml (all from Mulgrave, Australia, http://www.invitrogen.com) and fetal calf serum (FCS) 20% (Scoresby, Australia, http://www.hyclone.com/) at 37°C–5% CO_2_. Colonies were mechanically dissected every 7 days and transferred to freshly prepared MEFs. Media was changed every second day.

### PA6 Neural Induction

hESCs were mechanically dissected into pieces approximately 0.5 mm in diameter and transferred to an organ culture plate of PA6 cells (Koyadai, Japan, http://www.brc.riken.go.jp/lab/cell/) in coculture medium containing Glasgow minimum essential medium, supplemented with 8% knockout serum replacement, 1% NEAA, 2 mM l-glutamine, 1 mM sodium pyruvate, β-mercaptoethanol 0.1 mM, 2 mM, penicillin 25 U/ml, streptomycin 25 μg/ml. Cells were differentiated on PA6 cells for 12 days with Noggin (500 ng/ml, Minneapolis, MN, http://www.rndsystems.com) added to the media for the first 4 days. Following PA6 coculture neural rosettes were dissected into 0.5-mm fragments and further cultured in suspension in low-attachment 96-well plates (Mount Martha, Australia, http://www.corning.com) in N2B27 medium containing 1:1 mix of neuobasal medium with DMEM: nutrient mixture F-12 medium, supplemented with insulin/transferrin/selenium 1%, N2 1%, B27 1%, glucose 0.3%, penicillin 25 U/ml, and streptomycin 25 μg/ml (all from Invitrogen), as neurospheres with basic Fibroblast Growth Factor (bFGF) and Epidermal Growth Factor (EGF) (20 ng/ml each, R&D). After 7–10 days neurospheres were plated onto laminin-coated dishes in N2B27 medium without EGF and bFGF, supplemented with 1 mM dibutyryl-cAMP (Castle Hill, Australia, http://www.sigmaaldrich.com). For certain experiments SHH-N (200–1,000 ng/ml, R&D 1845-SH, C24II) was added to the coculture medium during neural induction.

### Neural Induction in Defined Medium

hESCs were mechanically dissected into pieces approximately 0.5 mm in diameter and transferred to laminin-coated organ culture plate in N2B27 medium for 12 days, with Noggin (500 ng/ml, R&D) added to the media only for the first 6 days followed by the addition of bFGF (20 ng/ml each, R&D) for the remaining 6 days. Following neural induction, the monolayer of neural progenitors were dissected into approximately 1 mm fragments and further cultured in suspension in low-attachment 96-well plates (Corning) in N2B27 medium supplemented with bFGF and EGF (20 ng/ml each, R&D). After 7–10 days neurospheres were plated onto laminin-coated dishes in N2B27 medium without EGF and bFGF, supplemented with 1 mM dibutyryl-cAMP (Sigma) for 7–10 days.

### Fluorescent Activated Cell Sorting Analysis

Neural stem cells (NSCs) were dissociated into single cells with TrypLE Express (Invitrogen) and fixed with 4% paraformaldehyde (PFA) for 10 minutes followed by premeabelization in 90% methanol. NSCs were immunolabeled with goat anti-FoxA2 (Santa Cruz, CA, http://www.scbt.com, dilution 1:300) or rabbit anti-TH (1:1000, Rogers, AR, http://www.pelfreez-bio.com) or mouse anti-TUJ1 (1:500, Sydney, Australia, http://www.promega.com) in blocking solution (Phosphate buffered saline-0.25% Triton X; PBT +10% FCS +10% FCS) for 60 minutes, and then were resuspended in appropriate Cy5 or Cy2 secondary antibodies (West Grove, PA, http://www.jacksonimmuno.com) for 30 minutes, followed by a wash in blocking solution before being immediately sorted using a FACSCalibur cell sorter.

### Immunostaining

Cell monolayers and neurospheres were fixed in 4% PFA for 20 minutes at 4°C and then washed briefly in Phosphate buffered saline (PBS). Neurospheres were embedded in Tissue-Tek OCT compound (Brendale, Australia, http://www.labtek.com.au), cut at 8 μm on a cryostat, and sections were placed on superfrost slides. Sections or culture dishes were blocked for 1 hour at room temperature (RT) in blocking solution. The following primary antibodies were used: goat anti-FoxA2 (1:300, Santa Cruz), mouse anti-Nkx2.1 (1:300, Cambridge, MA, http://www.abcam.com) goat anti-Sox2 (1:500, R&D), rabbit anti-Otx2 (1:1,000, North Ryde, NSW, Australia, http://www.millipore.com) rabbit anti-Corin (1:100; [28]), rabbit anti-TH (1:1,000, Pel Freez), mouse anti-Tuj1 (1:500, Promega), rabbit anti-Ki67 (prediluted, Thermoscientific), guinea pig anti-Lmx1b (1:5,000, gift Thomas Perlmann), mouse anti-Pax6 (1:40), mouse anti-Pax7 (1:40), mouse anti-En1 (1:40), and mouse anti-Nkx6.1 (1:10; all from Iowa City, Iowa http://www.dshb.biology.uiowa.edu). Antibodies were diluted in blocking solution incubated on sections overnight at 4°C. Following three 10-minute washes in PBT, the corresponding secondary antibodies were applied for 1 hour at RT: anti-goat Cy3, anti-mouse Cy3, ant-guinea pig Cy3, anti-rabbit Cy3, anti-rabbit Cy5, and anti-mouse Cy5 (1:400, Jackson Immunoresearch). Sections and cultures were counterstained for 10 minutes with Hoechst 33342 (5 μg/ml). Slides were mounted in PVA-DABCO for viewing under an immunofluorescent microscope (Mount Waverly, Australia, http://www.olympusaustralia.com.au), and images were captured using the Cell-M software. Confocal microscopy was performed using a North Ryde, Australia, http://www.zeiss.com.au or pascal.

### Reverse Transcription Polymerase Chain Reaction

Total RNA was isolated from cell pellets using the RNeasy Mini Spin Kit (Doncaster, Australia, http://www.qiagen.com) as per the manufacturer's instructions. RNA was quantified using a spectrophotometer, and 300 ng were transcribed using Superscript III (Invitrogen) as per the manufacturer's instructions. Negative controls were treated in the same fashion, but no Superscript III was added to the reaction mix. Standard polymerase chain reaction (PCR) was carried out using Taq DNA polymerase (Alexandria, Australia, http://www.bioline.com), gene-specific primers for SHH, dispatched homolog 1 (DISP1), and β-actin were designed (Supporting Information data; Hindmarsh, Australia, http://www.geneworks.com.au) and annealing temperatures of 55°C with amplification for 35 cycles. PCR products were resolved by agarose gel electrophoresis.

### Lentivirus Production and Neural Infection

293T cells were transfected by CaCl_2_ with the following plasmid combinations. pBR8.91 and pMDG.2 were mixed with 2K7-Ef1a-GFP-neo or 2K7-Ef1α-GLI1-IRES-EGFP-neo or RRL-Ef1α-GLI1-IRESGFP to generate lentiviral particles (see Supporting Information). Viral supernatant was harvested over 2 days and concentrated by ultracentrifugation. The concentrated lentiviral supernatant was titered on 293T cells to determine functional transducing units. NSCs were infected with 10 times the number of infectious particles per cells. Abbreviations: El1a, Elongation Factor 1 alpha; IRES, internal ribosome entry site; EGFP, enhanced green fluorescent protein.

### Statistical Method

The method used to quantify positive gene expression, as detected by immunostaining, was by using Image software program (http://www.scioncorp.com). Briefly, sections were photographed using an Olympus IX81 fluorescent microscope using the Cell-M software and/or a Zeiss LSM 510 meta or pascal confocal microscope using the LSM Zen software. Fluorescent objects were identified by the threshold function and evaluated by quantification of total number of pixels per section using Scion Image. The level of positive staining for gene of interest was measured by the same threshold and analyzed as a percentage of total pixels from the respective Hoechst image. Fluorescent Activated Cell Sorting (FACS) quantification of FOXA2, TH and β-III tubulin (TUJ1) were performed by analysis of at least 10,000 events per replicate. All experiments were repeated at least three times. One-way ANOVAs were performed for statistical analyses.

## RESULTS

### Neural Differentiation of hESCs on PA6 Yields Few Ventral Dopamine Neurons

**Figure 1:**
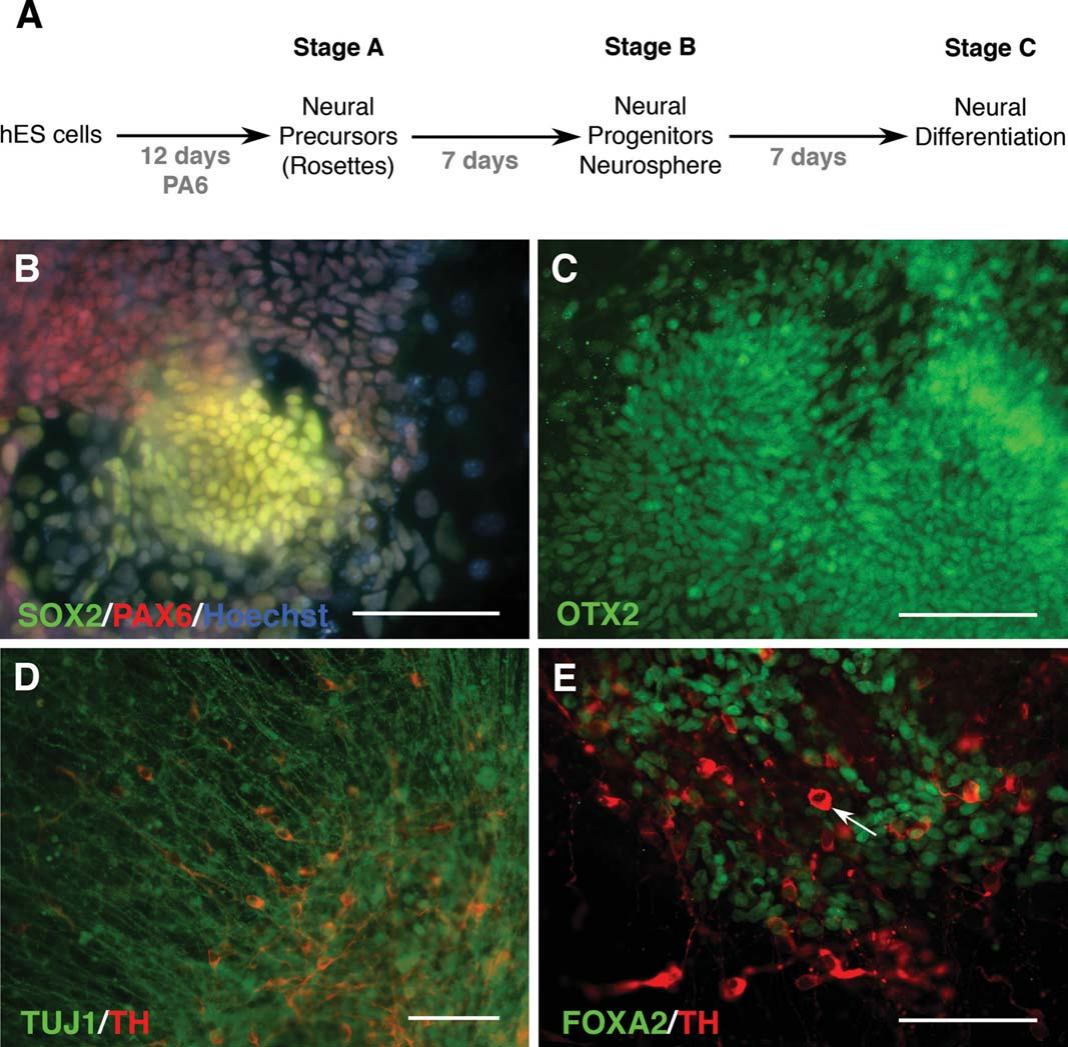
Few hESCs differentiate into ventral dopamine neurons. (**A**): hESCs are differentiated on PA6 cells for 12 days and form neural rosettes (stage A). Neural rosettes are dissected and cultured as floating spheres (stage B). Terminal differentiation of neurospheres results in neuronal differentiation (stage C). (**B**): Colocalization of PAX6 and SOX2 is observed during differentiation of hESCs to stage A. (**C**): Stage A neural rosettes express the forebrain marker OTX2. (**D**): Stage C neural cells express TUJ1 with a subset of cells colocalize for TH. (**E**): Few cells at stage C are positive for the ventral neural marker FOXA2+ and those which are not colocalize with the dopamine marker TH (arrow indicates TH+/FOXA2− cells). Scale bar = 100 μm. Abbreviations: hES, human embryonic stem; TH, tyrosine hydroxylase; TUJ1, β-III tubulin.

Differentiation of hESCs on the PA6 stromal cell line has been used extensively to direct their differentiation toward a NSC lineage. We have optimized a neural differentiation protocol utilizing PA6 cells supplemented with Noggin for the initial neural induction (stage A), neural expansion as neurospheres (stage B), and a final neuronal differentiation (stage C) on laminin. Stage A is characterized by the presence of neural rosettes that are immunopositive for pan-markers of early NSCs, SOX2 and PAX6 (Fig. 1B, 1C), as well as the forebrain-midbrain marker, OTX2 (Fig. 1D). These results are consistent with previously described hESC neural differentiation assays that yield a default rostral identity [29]. NSCs were then expanded as floating neurospheres (stage B) and further differentiated on laminin in the absence of exogenous growth factors to induce postmitotic neural differentiation (stage C), as indicated by the expression of the early postmitotic neuronal marker, TUJ1 (Fig. 1D). Further assessment of dopamine neuronal identity was conducted by identifying ventral dopamine neurons with the colocalization of FOXA2 and TH at stage C (Fig. 1E and Supporting Information Fig. S1D). It was rarely found that FOXA2 and TH colocalized (0.40% ± 0.29% SEM TH+/FOXA2+, Supporting Information Fig. S1D). This suggests using the PA6/Noggin system for hESC neural differentiation results in a low frequency of midbrain dopaminergic neurons.

### Recombinant SHH-N Suppresses PAX7 Dorsal Progenitors but Does Not Increase FOXA2 Ventral Progenitor Cells

**Figure 2:**
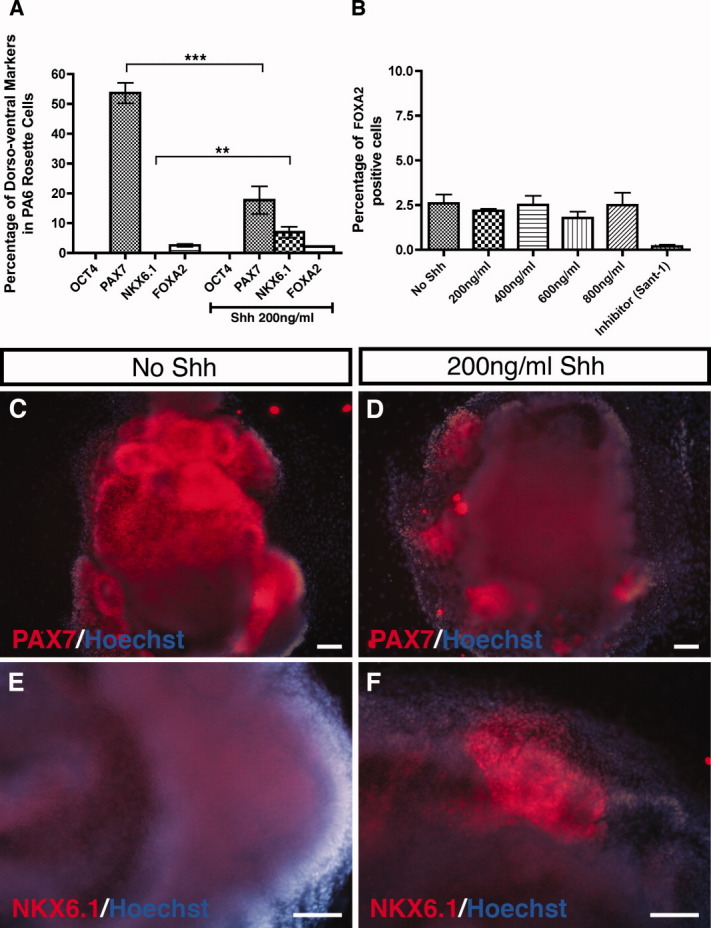
Recombinant SHH can suppress dorsal specification, but does not increase floor plate specification. (**A**): Bar graph showing percentages (±SEM) of stage A cells expressing the dorsoventral marks PAX7, NKX6.1 and FOXA2, and the pluripotent marker OCT4. Comparison of stage A cells treated with and without SHH-N (200 ng/ml). A significant decrease in PAX7 (*p* < .001) and a significant increase in NKX6.1 (*p* < .0045) is observed. (**B**): Bar graph shows various concentrations of SHH-N (200–800 ng/ml) do not significantly increase FOXA2 cells at stage A. However, a significant reduction in FOXA2 cells is seen with the inhibitor SANT-1 (*p* < .0092). (**C, D**): Immunofluorescence of PAX7 in colonies treated with and without SHH-N. (**E, F**): Immunofluorescence of NKX6.1 in colonies treated with and without SHH-N. Scale bar = 100 μm. Abbreviations: SHH, Sonic hedgehog; SHH-N, recombinant human N-terminal Sonic hedgehog.

The low frequency of dopaminergic neurons in the standard PA6/Noggin neural differentiation protocol may be due to a lack of the appropriate regionalization signals at early stages of neural induction/differentiation to bias the specification of neural progenitors to a ventral midbrain fate. To determine the dorsal-ventral identity of neural progenitors derived by PA6/Noggin neural induction, PAX7 expression was analyzed in neural rosettes (stage A). It was found that 53.66% ± 3.44% SEM of the colony expressed PAX7, suggesting a bias toward dorsal phenotype (Fig. 2A).

During embryogenesis, neuroepithelial cells in the ventral neural tube are specified by gradient levels of SHH released by the underlying notochord and FP. Thus, exogenous SHH has been used in several neural differentiation protocols to specify cells into a ventral lineage [23,30]. Addition of recombinant SHH-N at day1 of differentiation (stage A), at various doses, was assessed for its ability to generate FOXA2 expressing cells. Without SHH treatment, only 2.6% ± 0.49% SEM FOXA2 and 0.09% ± 0.06% SEM NKX6.1 were observed (Fig. 2A, 2E). When 200 ng/ml recombinant SHH-N was added from day 1, there was a significant decrease in PAX7 expression (*p* < .001; Fig. 2A, 2C, 2D) and a corresponding significant increase in NKX6.1 (*p* < .0045; Fig. 2A, 2E, 2F). However, no significant difference in FOXA2 was detected (Fig. 2A). A dose-response of SHH-N was conducted with up to 800 ng/ml of SHH-N and the levels of FOXA2 expression were again assessed. No significant increase in FOXA2 expression was detected, with even the highest amount of SHH-N added (Fig. 2B). However, a significant reduction in the number of FOXA2+ cells was observed when the cultures were treated with the SHH antagonist, SANT-1 (*p* < .0092; Fig. 2B). Thus, although exogenous treatment using recombinant SHH does not seem to be effective in increasing the proportion of FOXA2+ cells, SHH is required for inducing FOXA2 expression.

### Neural Differentiation of hESCs in Defined Medium Generates Early Rosettes Before Dorsoventral Commitment

**Figure 3:**
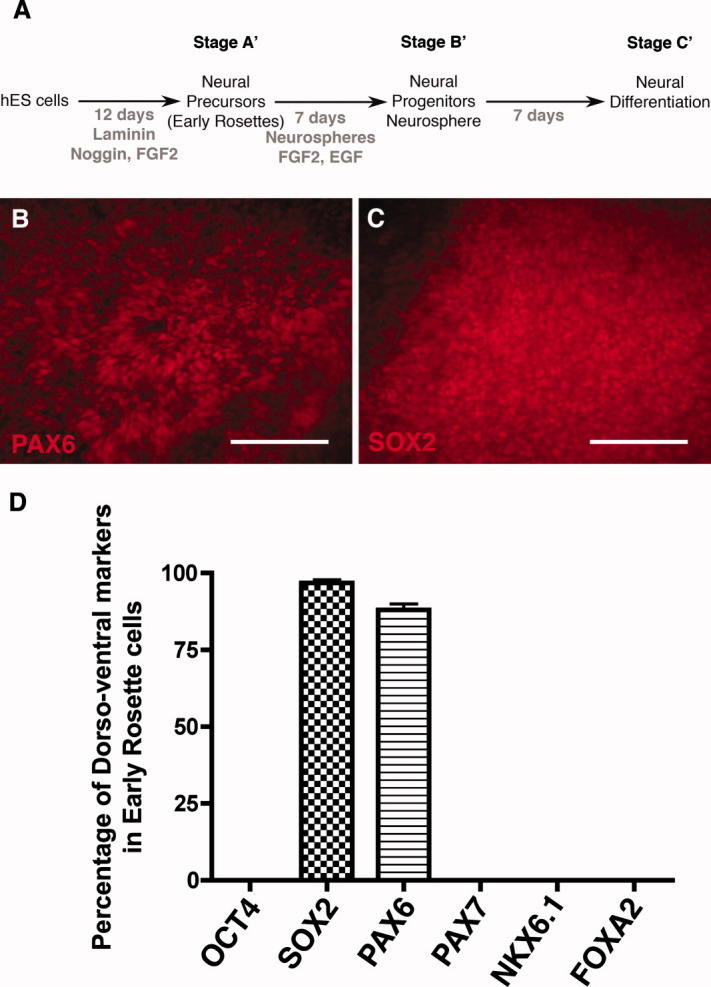
Differentiation of hESCs in defined media with Noggin results in uncommitted neuroepithelial cells, which can further differentiate into neurons. (**A**): hESCs are differentiated on laminin in defined medium for 12 days and form early neural rosettes (stage A′). Neural rosettes are dissected and cultured as floating spheres (stage B′). Terminal differentiation of neurospheres results in neuronal differentiation (stage C′). (**B, C**): Stage A′ neural cells express PAX6 and SOX2. (**D**): Bar graph shows at stage A′ early neural rosettes express no dorsoventral markers and do not express the pluripotent stem cell marker OCT4, as assessed by immunofluorescence. In contrast, 96.75% (±1.031 SEM) and 87.96% (±2.063 SEM) of cells express the neuroepithelial stem cell marker SOX2 and PAX6, respectively. Scale bar = 100 μm. Abbreviations: EGF, Epidermal Growth Factor; FGF2, Fibroblast Growth Factor 2; hES, human embryonic stem.

The aforementioned data showed that when using PA6 coculture for hESC neural induction, the onset of PAX7 expression was detected during the neural induction stage, stage A. This suggests that PA6 may differentiate hESCs to specified neural precursors rapidly and thus, for temporal reasons, it is difficult for cells to be responsive to factors supplemented within the medium. We therefore sought to develop a differentiation system in the absence of PA6 to have a temporal window of uncommitted early neural precursors. hESCs were plated on laminin in defined N2B27 medium supplemented with Noggin for 12 days (Fig. 3A; stage A′) and then assessed for dorsalventral neural patterning markers. At stage A′, OCT4 is no longer expressed and cultures consist of PAX6+ and SOX2+ rosette-forming cells. In addition, there is no detected expression of regional markers PAX7, NKX6.1, and FOXA2 (Fig. 3D). These features taken together are characteristic of early neural precursors [31]. Using this neural induction protocol, SHH at 200 ng/ml or 1,000 ng/ml was supplemented in the media and levels of FOXA2 expression was analyzed (Supporting Information Fig. S2). It was found that, similar to the PA6 coculture neural induction system, only on rare occasions are FOXA2 cells detected at stage A′ even in the presence of high levels (1,000 ng/ml) of recombinant SHH. This data suggest that the default pathway of neural induction and differentiation results in a low population of FOXA2 cells and additional extrinsic and/or intrinsic signals are required to specify hESC-derived neural precursors to a ventral fate.

### Misexpression of GLI1 Induces FP Specification of NSCs

Given the lack of effectiveness for recombinant SHH to induce FOXA2 expression, an alternative approach of using intrinsic signals was undertaken to specify hESC-derived neural precursors to a ventral fate. The candidate intrinsic signal chosen was the transcription factor GLI1. Gli1 is initially coexpressed with FoxA2 cells in the embryonic FP region of the neural tube and is a direct downstream target of Shh [7]. Additionally, Gli1 does not contain an N-terminal repressor domain, which in the case of Gli2 and Gli3 can be activated after cleavage [32].

**Figure 4:**
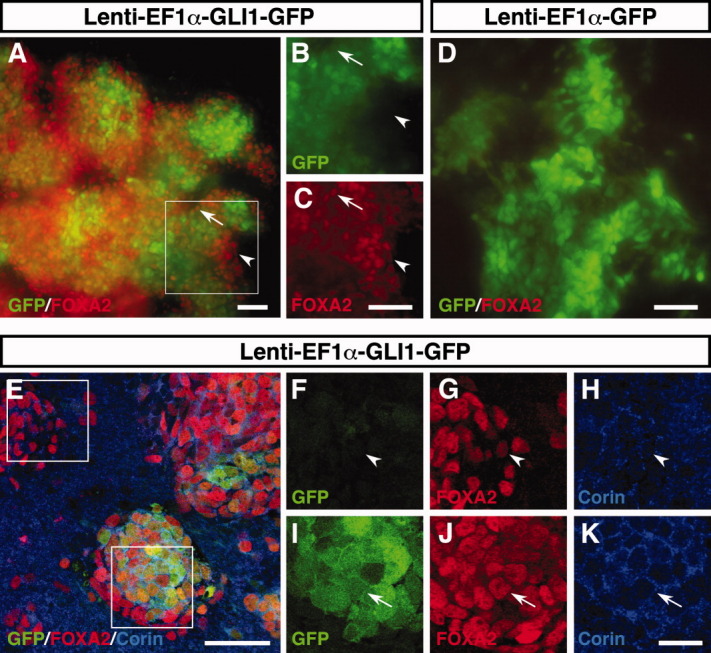
Misexpression of GLI1 in stage A′ cells results in upregulation of FOXA2 and the floor plate marker Corin. (**A–C**): GLI1-IRES GFP lentivirus induces FOXA2 expression in infected cells (arrow) and GFP-/FOXA2+ cells can also be found (arrowhead). (**D**): Control GFP lentivirus does not induce FOXA2 expression. (**E–K**): Double-positive FOXA2 and Corin cells are observed in the GLI1-IRES GFP-infected cells. Some of the FOXA2+/Corin+ cells showed low or no expression of GFP ([**F–H**], arrowhead), however, several GLI1-IRES GFP-infected cells showed to be double-positive FOXA2/Corin ([**I–K**], arrow). Scale bar = 50 μm (**A–E**), 20 μm (**F–K**). Abbreviations: EF1α, Elongation Factor 1 α; GFP, Green Fluorescent Protein.

**Figure 5:**
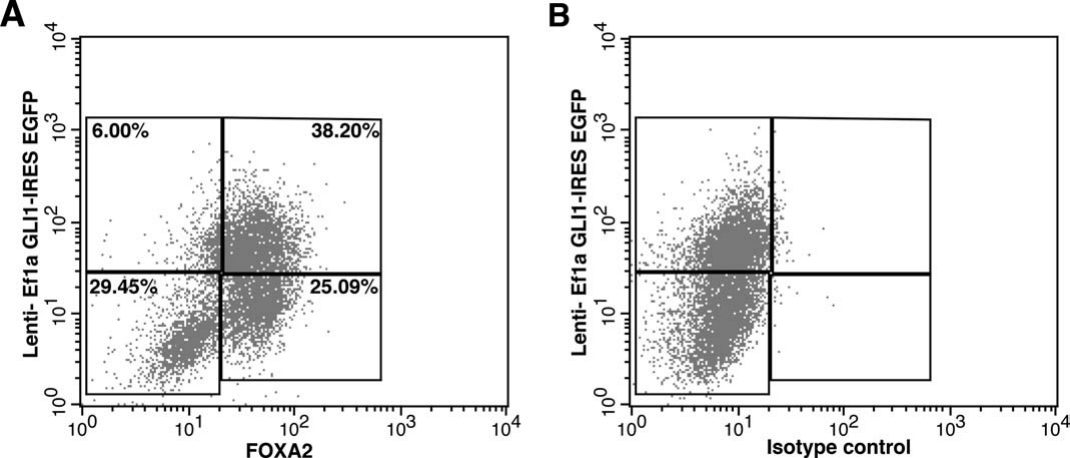
FACS analysis of GLI1-transduced colonies reveal FOXA2-positive cells, which do not coexpress GFP. (**A**): FACS analysis of stage A′ cells infected with GLI1-IRES-GFP lentivirus showed 38.2% (±4.40 SEM) FOXA2+/GFP+ and 25.09% (±1.79 SEM) FOXA2+/GFP− cells. (**B**): Isotype control FACS. Abbreviations: Ef1a, Elongation Factor 1 α; EGFP, Enhanced Green Fluorescent Protein; IRES, Internal ribosome entry site.

Using lentiviral technology, hESC-derived neural precursors were infected at stage A′ with a GLI1-IRES-EGFP virus driven by a constitutively active promoter. At 12 days after infection with GLI-IRES-EGFP, 63.27% ± 4.85% SEM cells were FOXA2+ (Figs. 4A–4C, 5). Coexpression of the ventral neural tube marker, Corin, and Green Fluorescent Protein (GFP) were also observed (Fig. 4E–4K). In addition, 11.76% of cells within the cultures expressed the SHH-responsive and basal plate marker NKX6.1 (data not shown). Neural progenitors infected with control virus (EGFP), resulted in no detectable FOXA2 expression (Fig. 4D). Thus, similar to the embryonic neural tube, GLI1 expression induces FOXA2 expression and ventralizes neural progenitors.

**Figure 6:**
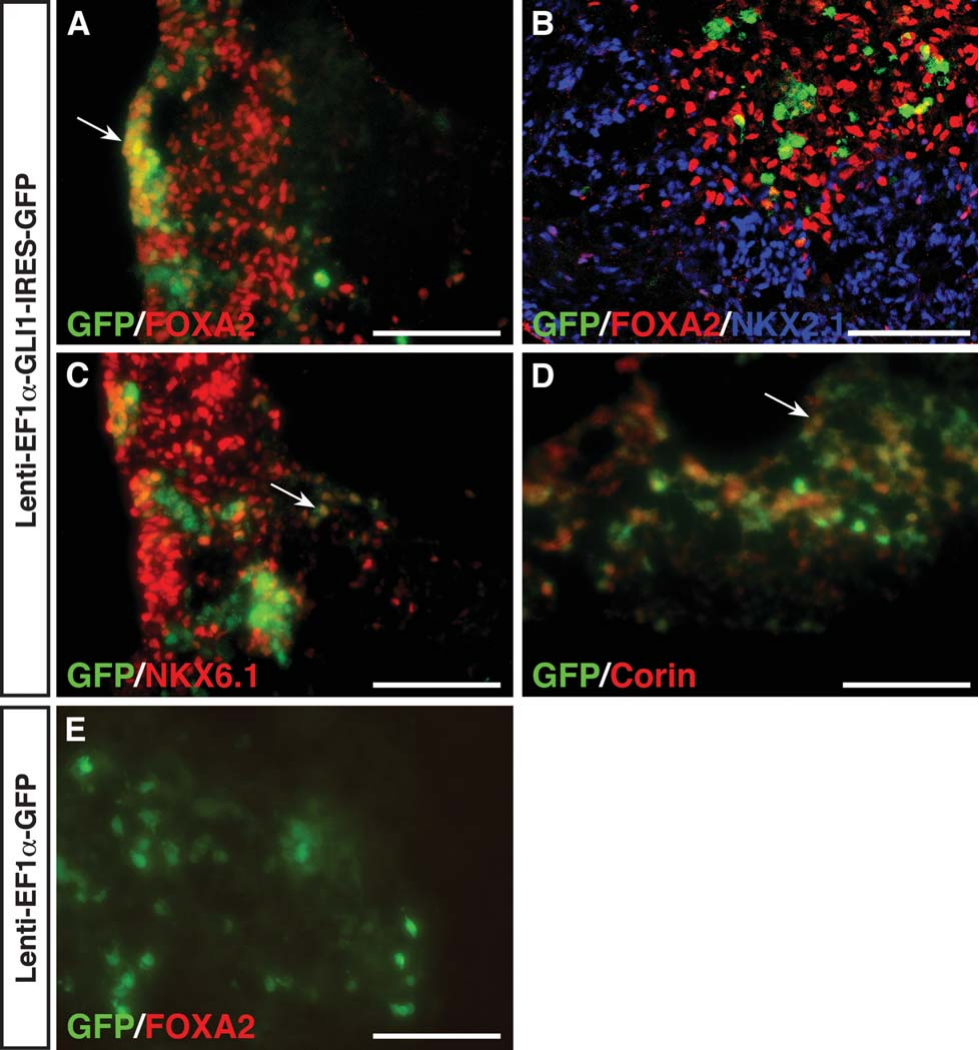
GLI1-transduced stage A′ neural cells form neurospheres (stage B′), which maintain floor plate and ventral neural stem cell markers. (**A**): GLI1-infected cells maintain FOXA2 expression in neurospheres and FOXA2+/GFP− cells can be seen in close proximity to FOXA2+/GFP+ cells (arrow). (**B**): The GFP+/FOXA2+ did not colocalize with the ventral forebrain marker, NKX2.1. (**C**): A small proportion of GFP+ cells coexpress NKX6.1+ (arrow). NKX6.1+/GFP− cells are also seen in the GLI1-infected neurospheres. (**D**): Corin+/GFP+ cells can be identified within the neurospheres (arrow), along with Corin−/GFP+ cells and Corin+/GFP− cells. (**E**): Cells infected with GFP control lentivirus did not show any GFP+/FOXA2+ cells. Scale bar = 100 μm. Abbreviations: EF1α, Elongation Factor 1 α; GFP, Green Fluorescent Protein; IRES, Internal ribosome entry site.

Interestingly, in the GLI1-transduced cultures 25.09% ± 1.79% SEM FOXA2+ cells did not coexpress GFP (Fig. 5). The FOXA2+/GFP- population may arise from transduced cells that subsequently silenced GFP expression. Alternatively, or perhaps in addition to, the FOXA2+/GFP− cells may arise from local signals secreted by the GFP+ cells, namely, SHH, somewhat mimicking the embryonic neural tube FP. Indeed, this latter hypothesis is supported by the observation that the GLI1-tranduced cultures show the presence of NKX6.1+/GFP− cells, which was undetected in noninfected controls (Fig. 6C).

To determine the mechanism by which FOXA2+/GFP− cells were derived from, coculture experiments were performed whereby a constitutively expressing GFP cell line, ENVY was infected with GLI1-IRES-GFP. Following infection, ENVY cells were harvested and then cocultured with a non-GFP hESC line, HES-3, for 12 days after which FOXA2+/GFP− cells were identified (Supporting Information Fig. S3). These results support the scenario that GLI1-expressing cells are influencing the neighboring cells to also be ventralized. Overall, these data demonstrates that GLI1 is a potent factor for ventralizing neural progenitors, by mediating its function both intrinsically and extrinsically.

### GLI1-Transduced Cells Secrete SHH

GLI1-transduced neural progenitors were further differentiated to neurospheres and the expression of dorsalventral regional markers were analyzed at this stage of differentiation, stage B′. GFP expression persisted at the neurosphere stage, and again, it overlapped with FOXA2 expression (Fig. 6). Similar to stage A′, there were FOXA2+/GFP− cells present (Fig. 6A), as well as expression of Corin (Fig. 6D), NKX6.1 (Fig. 6C), and NKX2.1 (Fig. 6B).

The mechanisms by which GLI1 is inducing expression of ventral markers in neighboring cells may be through its secretion of SHH, as previous studies have suggested in other species [33,34]. Reverse transcription polymerase chain reaction (RT-PCR) was performed on neurospheres derived from GLI-transduced progenitors and control GFP-transduced progenitors. SHH transcripts were detected in neurospheres derived from GLI1-transduced progenitors only (Supporting Information Fig. S4), further supporting the FP-like activity of GLI1-expressing neural progenitors.

### High Levels of FOXA2 Expression is Maintained on Terminal Differentiation of Neurospheres Derived from GLI1-Transduced Cells

Expression of Ki67 was analyzed in neurospheres derived from GLI1-transduced cells to determine whether the Gli1+/GFP+ cells are able to differentiate into postmitotic progenitors (Supporting Information Fig. S5). Both populations of GFP+/Ki67+ and GFP+/Ki67− cells were observed suggesting that GFP+ cells are capable of terminal differentiation.

**Figure 7:**
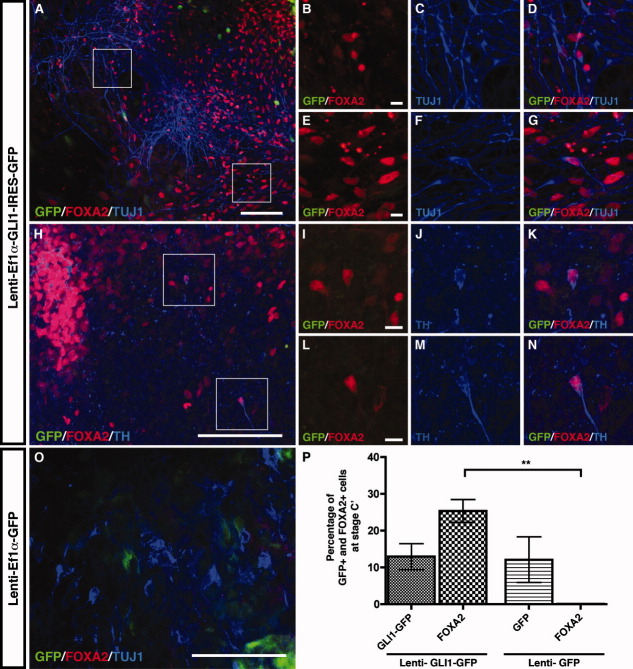
GLI1-transduced neural stem cells can differentiate into neurons (stage C′) and express dopamine marker, TH. (**A–N**): Both FOXA2+/TUJ1+ cells (**A–G**) and FOXA2+/TH+ cells (**H–N**) are found in the differentiated GLI1-transduced cultures. (**O**): Control GFP lentivirus infected cultures differentiate into neurons with a subset expressing TH, however, no cells colocalized with FOXA2. (**P**): Bar graph showing percentage of cells expressing both GFP and FOXA2 within the GLI1-infected cells and GFP lentiviral control cells at stage C′. There was a significant increase (*p* < .0067) in FOXA2 expression in GLI1-transduced cells 25.37% (±3.106 SEM) FOXA2+ cells compared with GFP control lentiviral cultures 0.08% (±0.076 SEM) FOXA2+ cells. Scale bar = 100 μm (**A, H, O**), 10 μm (**B–G, I–N**). Abbreviations: Ef1α, Elongation Factor 1 α, IRES, Internal ribosome entry site; GFP, Green Fluorescent Protein; TH, tyrosine hydroxylase; TUJ1, β-III tubulin.

Neurospheres derived from GLI1-expressing cells were then further differentiated onto laminin in the absence of mitogens to promote their differentiation to postmitotic neurons and glia (stage C′). At stage C′, it was found that the levels of GFP+ expression drops to 12.94% ± 3.551% SEM, as compared with 44.2% ±4.044% SEM at stage A′ (Figs. 5, 7P). This supports the notion of silencing of the transgene over time and on differentiation. Despite this significant (*p* < .0022) decrease in GFP expression, the levels of FOXA2 maintained to be significantly high (25.37% ± 3.106% SEM; *p* < .0067; Fig. 7P). Several FOXA2 cells also coexpressed β-tubulin (Fig. 7A–7G), and 1.63% ±0.24% SEM were FOXA2+/TH+ (Fig. 7H–7N and Supporting Information Fig. S1). The low levels of FOXA2+/TH+ expression found in the differentiated GLI1-transduced cultures suggests a lack of midbrain specification and perhaps instead these cultures are fated toward a more anterior identity. In support of this, very few LMX1B+ and Engrailed 1+, both mesDA neural progenitor markers, were detected in the GLI1-transduced cultures at stages B′ and C′ (Supporting Information Fig. S6). In all, these data demonstrates that GLI1 is a potent inducer of FOXA2 expression in neural precursors and consequently may therefore bias their fate toward ventral neurons of the neural tube.

## DISCUSSION

Differentiation of hESC into specific neuronal lineages requires having precise temporal and spatial patterning signals, similar to embryonic neural development, to commit their differentiation toward certain cell fates and prevent their differentiation down alternate lineages. SHH is a key morphogen in establishing a dorsoventral neural patterning within the embryonic neural tube and at high concentrations specifies the ventral midline cells to a FP fate [14]. In this study, we showed that human recombinant SHH treatment of hESC during neural induction is capable of inhibiting a dorsal neural fate and promotes expression of the basal neural tube marker, NKX6.1. However, despite having a shift to more ventral cell types, there was no significant increase in FOXA2 expression, even when high concentrations of recombinant SHH were used. One possible explanation is that the PA6 feeder layer does not allow high levels of SHH signaling to occur, due to other inhibiting factors. Indeed, Fasano et al. [24] showed FP specification by combining the Dkk1 inhibitor together with mouse recombinant SHH, suggesting that other cofactors may enhance ventralization of the neural progenitors. Alternatively, or perhaps in addition to, the effectiveness of SHH on promoting FP fate in neural progenitors may be temporally dependent. In the embryo, SHH released by the notochord induces FP development which in turn establishes a SHH gradient for patterning the basal plate of the neural tube. Thus, inducing a FP identity requires early and high levels of SHH exposure during neural induction. Using the PA6 coculture system, expression of neural patterning factors were detected relatively early, at the neural induction stage (stage A), suggesting an early onset of commitment to neuronal identity. Indeed, mouse DA specification has also been shown to occur early during differentiation on PA6 cells [17]. Thus, a modified culture system was established to remove the PA6 feeder layer and also to allow a longer temporal window whereby hESC-derived neural progenitors show no expression of dorsoventral regional markers. Using this defined culture system, we again could not induce significant proportions of FOXA2-positive cells when exposed to recombinant SHH.

SHH is endogenously synthesized as a full-length precursor that undergoes autoproteolytical cleavage. The final N-terminal product has a cholesterol group added to its C-terminus and a palmitoyl group to the N-terminus. Both lipid modifications affect the range and potency of SHH action, thereby also regulating its morphogenic gradient. One of these factors is the ability of the lipid-modified SHH to form an oligomer [35]. The lipid-modified SHH can only be secreted via its interaction with DISP1 [36]. Commercially available recombinant SHH proteins are not lipid-modified and are therefore less potent in their activity due to their inability to form oligomers [35,36]. During the review period of our study, Fasano et al. described a more potent, modified version of recombinant mouse SHH (C25II) which showed to induce a FP phenotype in neural progenitors derived from hESC [24]. In vivo secretion of N-terminal SHH protein, although can partially rescue the phenotype of SHH null mice, there is still an observed contraction of the dorsoventral cell types found within the neural tube [37]. This contraction is attributed to the failure for nonlipid-modified SHH to accumulate on the apical surface of cells and a reduction of SHH activity in its monomeric form. These data indicates that SHH potency is far less effective when administered exogenously, adding to the notion that high levels of SHH are required for ventral cell type specification.

The insufficient potency of using recombinant SHH, as described earlier, led us to explore an alternative approach to intrinsically specify hESC-derived neural progenitors cells to a FP fate. SHH acts upstream of FOXA2, which then further induces the expression of SHH, creating a positive feedback cycle. GLI1 is also a downstream target of SHH signaling and is known to upregulate FOXA2 expression. Previous studies in mice showed that forced Gli1 expression in the dorsal neural tube results in ectopic FoxA2 expression [15], suggesting that Gli1 may be an appropriate candidate for inducing FP fate in neural progenitors. Our data show that GLI1 was capable of ventralizing hESCs-derived early neural rosettes into FP cells as indicated by the coexpression of FOXA2 and Corin. More importantly, neurospheres derived from GLI1/GFP-transduced cultures showed a significant increase in GFP−/FOXA2+ and GFP-/NKX6.1+ cells, which were observed to be in close proximity to the GFP+/GLI1+ cells. This effect may be due to localized SHH released from GLI1/GFP-transduced cells and/or silencing of the GLI1/GFP construct. The coculture experiments, using GLI1-transduced ENVY hESCs (that constitutively expresses GFP) cocultured with non-GFP HES-3, also showed localized upregulated expression of FOXA2 in non-GFP expressing cells, thereby supporting the former hypothesis. RT-PCR analyses detected SHH transcripts in GLI1-transduced cultures, further suggesting a paracrine effect of GLI1-expressing progenitors to secrete SHH, indicative of FP action *in vivo*. Furthermore, SHH produced by the GLI1-expressing cells would presumably be secreted in the lipid-modified oligomeric form and thus act more potently than exogenous administration of nonlipid-modified recombinant SHH. Taken together, these data show that the GLI1-expressing cells mechanistically recapitulate the neural tube FP.

The timing of GLI1 expression in neural progenitors is also critical for inducing a FP. In the mouse, only an early (E7.5–E8.5) transient population of Gli1-expressing cells in the FP region of the neural tube have been shown to give rise to mesDA neurons [7]. At later stages of development, Gli1 is expressed in more basal regions of the neural tube and are no longer fated to FP. These results highlight the importance of inducing Gli1 expression in uncommitted neural progenitors at early stages during their differentiation to promote their specification toward a FP lineage, which then later may give rise to ventral neural progenitors including mesDA neurons.

Our data showed low levels of midbrain neural patterning markers in the differentiated GLI1-transduced cultures. This suggests that, in addition to ventralizing factors, anterior/posterior specifying factors are required to generate mesDA neural progenitors. For example, addition of exogenous Fibroblast Growth Factor 8 (FGF8) and Wnt1 into culture systems have extensively been used to spatially pattern the anterior-posterior axis of cells into a midbrain region [22,29,38,39]. Wnt1 has also been shown to directly regulate Lmx1a with an autoregulatory loop being formed, which then regulates Pitx3 and Nurr1 expression [39,40]. Furthermore, the SHH/FoxA2 pathway positively regulates Nurr1 and Ngn2 expression and inhibits Nkx2.2 [40,41]. The interaction of these two pathways combined are critical in the efficient production of mDA neurons from hESCs and the inhibition of alternate cell fates [24]. Our data shows GLI1 as a determinant in activating the SHH/FOXA2 pathway by generating FP cells.

## CONCLUSION

Our research demonstrated that proper ventralization of neurons prior to commitment into a dorsoventral identity is essential in generating FP cells. We have shown that the differentiation of induced FP cells by forced GLI1 expression can give rise to ventral DA neurons, which is an important step in generating a homogeneous population of mesDA neurons. In summary, this study describes the generation of FP cells from hESC using a single intrinsic factor, GLI1, which may serve as a foundation for deriving various ventral neuronal populations, including mesDA for treating Parkinson's disease.

## DISCLOSURE OF POTENTIAL CONFLICTS OF INTEREST

The authors indicate no potential conflicts of interest.

## References

[1] Perrier AL, Tabar V, Barberi T (2004). Derivation of midbrain dopamine neurons from human embryonic stem cells. Proc Natl Acad Sci USA.

[2] Cho MS, Lee YE, Kim JY (2008). Highly efficient and large-scale generation of functional dopamine neurons from human embryonic stem cells. Proc Natl Acad Sci USA.

[3] Friling S, Andersson E, Thompson LH (2009). Efficient production of mesencephalic dopamine neurons by Lmx1a expression in embryonic stem cells. Proc Natl Acad Sci USA.

[4] Riddle R, Pollock JD (2003). Making connections: The development of mesencephalic dopaminergic neurons. Brain Res Dev Brain Res.

[5] Wallen A, Zetterstrom RH, Solomin L (1999). Fate of mesencephalic AHD2-expressing dopamine progenitor cells in NURR1 mutant mice. Exp Cell Res.

[6] Kawano H, Ohyama K, Kawamura K (1995). Migration of dopaminergic neurons in the embryonic mesencephalon of mice. Brain Res Dev Brain Res.

[7] Zervas M, Millet S, Ahn S (2004). Cell behaviors and genetic lineages of the mesencephalon and rhombomere 1. Neuron.

[8] Kittappa R, Chang WW, Awatramani RB (2007). The foxa2 gene controls the birth and spontaneous degeneration of dopamine neurons in old age. Plos Biol.

[9] Bonilla S, Hall AC, Pinto L (2008). Identification of midbrain floor plate radial glia-like cells as dopaminergic progenitors. Glia.

[10] Nelander J, Hebsgaard JB, Parmar M (2009). Organization of the human embryonic ventral mesencephalon. Gene Expr Patterns.

[11] Ang SL, Rossant J (1994). HNF-3 beta is essential for node and notochord formation in mouse development. Cell.

[12] Chiang C, Litingtung Y, Lee E (1996). Cyclopia and defective axial patterning in mice lacking Sonic hedgehog gene function. Nature.

[13] Yamada T, Placzek M, Tanaka H (1991). Control of cell pattern in the developing nervous system: polarizing activity of the floor plate and notochord. Cell.

[14] Echelard Y, Epstein DJ, St-Jacques B (1993). Sonic hedgehog, a member of a family of putative signaling molecules, is implicated in the regulation of CNS polarity. Cell.

[15] Hynes M, Stone DM, Dowd M (1997). Control of cell pattern in the neural tube by the zinc finger transcription factor and oncogene Gli-1. Neuron.

[16] Schulz TC, Noggle SA, Palmarini GM (2004). Differentiation of human embryonic stem cells to dopaminergic neurons in serum-free suspension culture. Stem Cells (Dayton, Ohio).

[17] Parmar M, Li M (2007). Early specification of dopaminergic phenotype during ES cell differentiation. BMC Dev Biol.

[18] Iacovitti L, Donaldson AE, Marshall CE (2007). A protocol for the differentiation of human embryonic stem cells into dopaminergic neurons using only chemically defined human additives: Studies in vitro and in vivo. Brain Res.

[19] Cai J, Donaldson A, Yang M (2009). The role of Lmx1a in the differentiation of human embryonic stem cells into midbrain dopamine neurons in culture and after transplantation into a Parkinson's disease model. Stem Cells (Dayton, Ohio).

[20] Lee SH, Lumelsky N, Studer L (2000). Efficient generation of midbrain and hindbrain neurons from mouse embryonic stem cells. Nat Biotechnol.

[21] Mizuseki K, Sakamoto T, Watanabe K (2003). Generation of neural crest-derived peripheral neurons and floor plate cells from mouse and primate embryonic stem cells. Proc Natl Acad Sci USA.

[22] Sonntag KC, Pruszak J, Yoshizaki T (2007). Enhanced yield of neuroepithelial precursors and midbrain-like dopaminergic neurons from human embryonic stem cells using the bone morphogenic protein antagonist noggin. Stem Cells (Dayton, Ohio).

[23] Kim JH, Auerbach JM, Rodriguez-Gomez JA (2002). Dopamine neurons derived from embryonic stem cells function in an animal model of Parkinson's disease. Nature.

[24] Fasano CA, Chambers SM, Lee G (2010). Efficient derivation of functional floor plate tissue from human embryonic stem cells. Cell Stem Cell.

[25] Cowan CA, Klimanskaya I, McMahon J (2004). Derivation of embryonic stem-cell lines from human blastocysts. N Engl J Med.

[26] Reubinoff BE, Pera MF, Fong CY (2000). Embryonic stem cell lines from human blastocysts: Somatic differentiation in vitro. Nat Biotechnol.

[27] Conley BJ, Denham M, Gulluyan L (2005). Mouse embryonic stem cell derivation, and mouse and human embryonic stem cell culture and differentiation as embryoid bodies. Curr Protoc Cell Biol.

[28] Jonsson ME, Ono Y, Bjorklund A (2009). Identification of transplantable dopamine neuron precursors at different stages of midbrain neurogenesis. Exp Neurol.

[29] Denham M, Dottori M (2009). Signals involved in neural differentiation of human embryonic stem cells. Neurosignals.

[30] Andersson E, Tryggvason U, Deng Q (2006). Identification of intrinsic determinants of midbrain dopamine neurons. Cell.

[31] Lee H, Shamy GA, Elkabetz Y (2007). Directed differentiation and transplantation of human embryonic stem cell-derived motoneurons. Stem Cells (Dayton, Ohio).

[32] Sasaki H, Nishizaki Y, Hui C (1999). Regulation of Gli2 and Gli3 activities by an amino-terminal repression domaimplication of Gli2 and Gli3 as primary mediators of Shh signaling. Development (Cambridge, England).

[33] Marti E, Bumcrot DA, Takada R (1995). Requirement of 19K form of Sonic hedgehog for induction of distinct ventral cell types in CNS explants. Nature.

[34] Sasaki H, Hui C, Nakafuku M (1997). A binding site for Gli proteins is essential for HNF-3beta floor plate enhancer activity in transgenics and can respond to Shh in vitro. Development (Cambridge, England).

[35] Chen MH, Li YJ, Kawakami T (2004). Palmitoylation is required for the production of a soluble multimeric Hedgehog protein complex and long-range signaling in vertebrates. Genes Dev.

[36] Burke R, Nellen D, Bellotto M (1999). Dispatched, a novel sterol-sensing domain protein dedicated to the release of cholesterol-modified hedgehog from signaling cells. Cell.

[37] Huang X, Litingtung Y, Chiang C (2007). Region-specific requirement for cholesterol modification of sonic hedgehog in patterning the telencephalon and spinal cord. Development (Cambridge, England).

[38] Castelo-Branco G, Wagner J, Rodriguez FJ (2003). Differential regulation of midbrain dopaminergic neuron development by Wnt-1, Wnt-3a, And Wnt-5a. Proc Natl Acad Sci USA.

[39] Chung S, Leung A, Han BS (2009). Wnt1-lmx1a forms a novel autoregulatory loop and controls midbrain dopaminergic differentiation synergistically with the SHH-FoxA2 pathway. Cell Stem Cell.

[40] Ferri AL, Lin W, Mavromatakis YE (2007). Foxa1 and Foxa2 regulate multiple phases of midbrain dopaminergic neuron development in a dosage-dependent manner. Development (Cambridge, England).

[41] Lin W, Metzakopian E (2009). Foxa1 and Foxa2 function both upstream of and cooperatively with Lmx1a and Lmx1b in a feedforward loop promoting mesodiencephalic dopaminergic neuron development. Dev Biol.

