# Supplementary figures and images for: Gli1 Is an Inducing Factor in Generating Floor Plate Progenitor Cells from Human Embryonic Stem Cells
Source: Stem Cells. 2010 Aug 26;28(10):1805–15. doi: 10.1002/stem.510 (PMC2996857; doi:10.1002/stem.510)

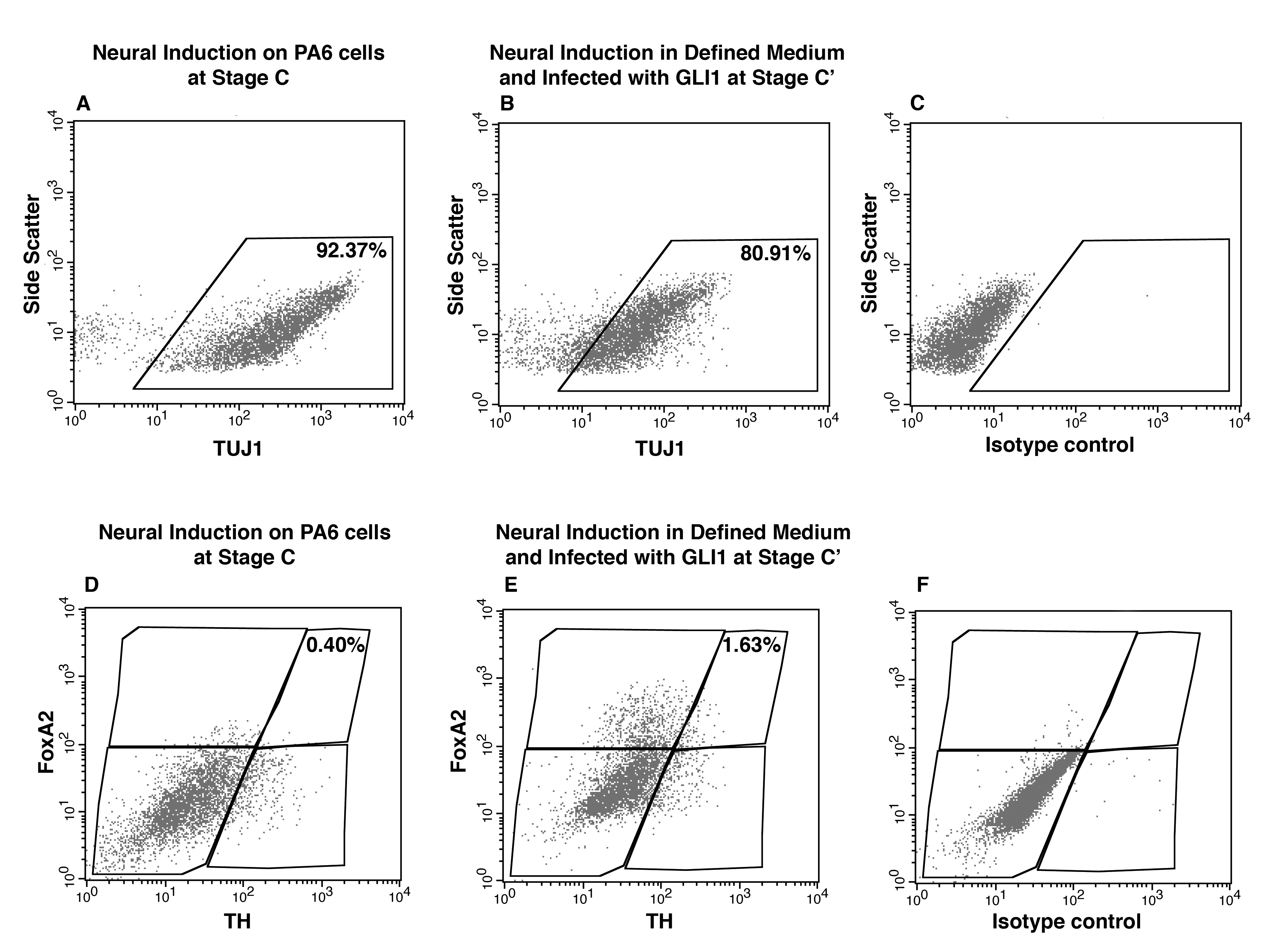

Supplement: Supplementary file 1 [file stem0028-1805-SD1.tif]

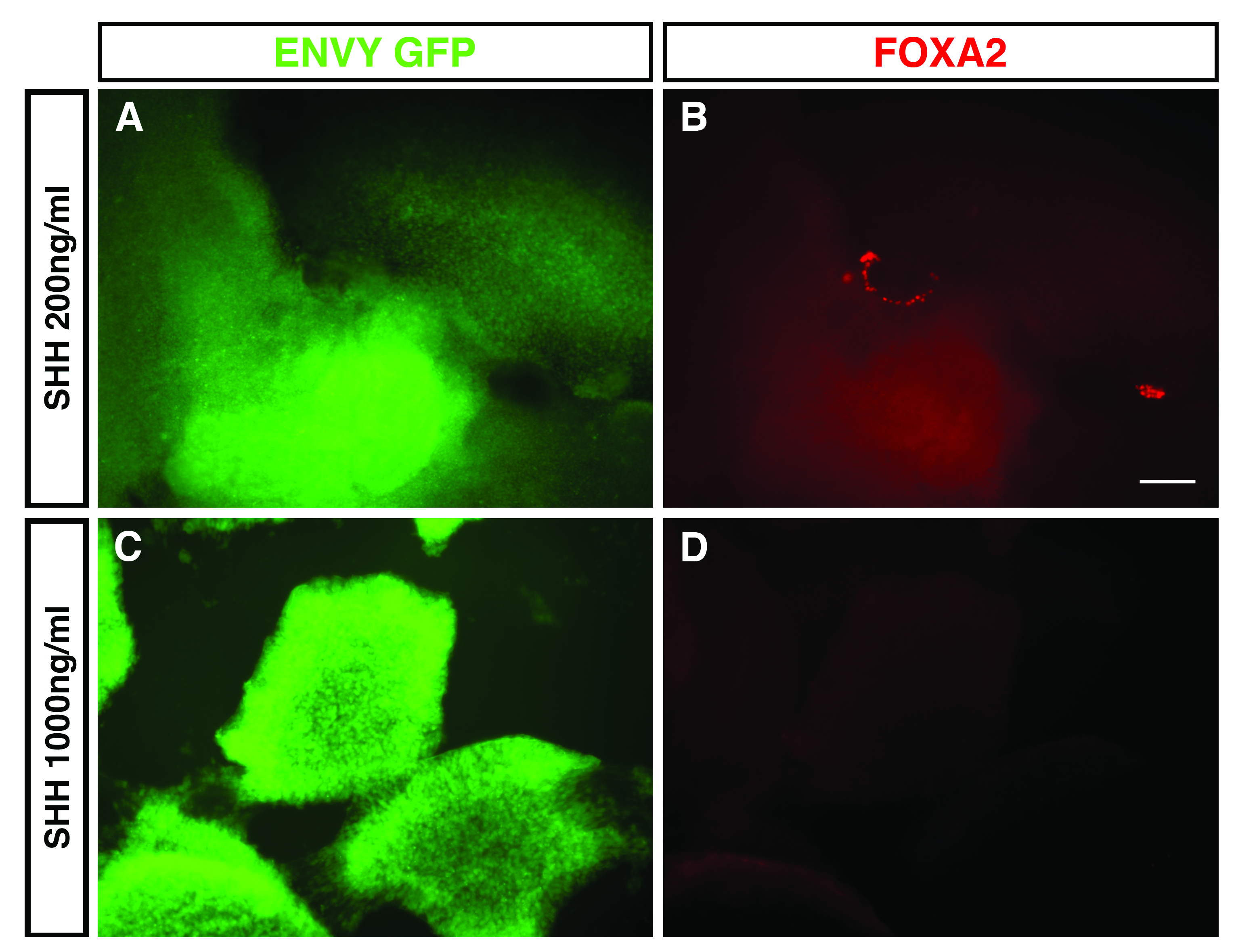

Supplement: Supplementary file 2 [file stem0028-1805-SD2.tif]

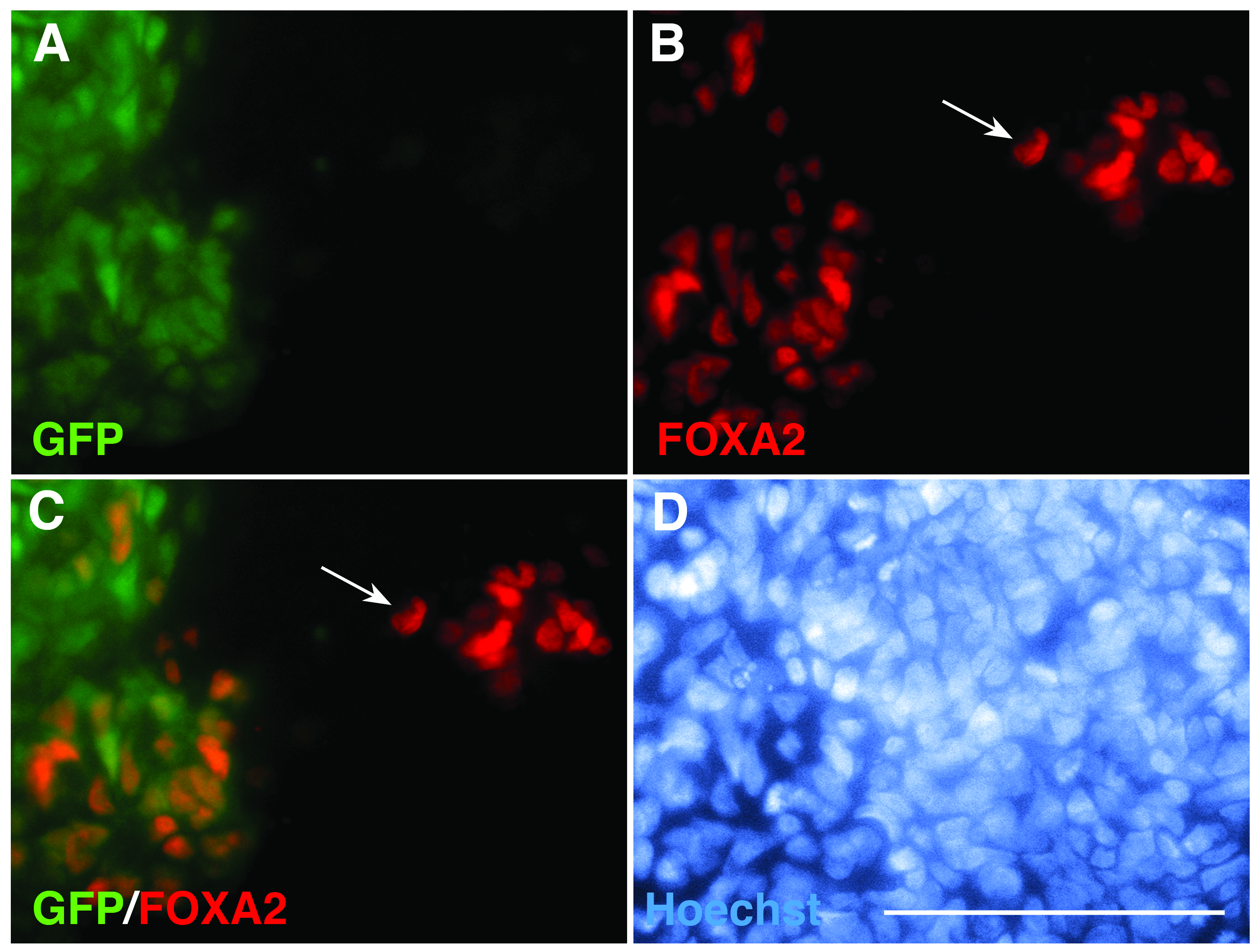

Supplement: Supplementary file 3 [file stem0028-1805-SD3.tif]

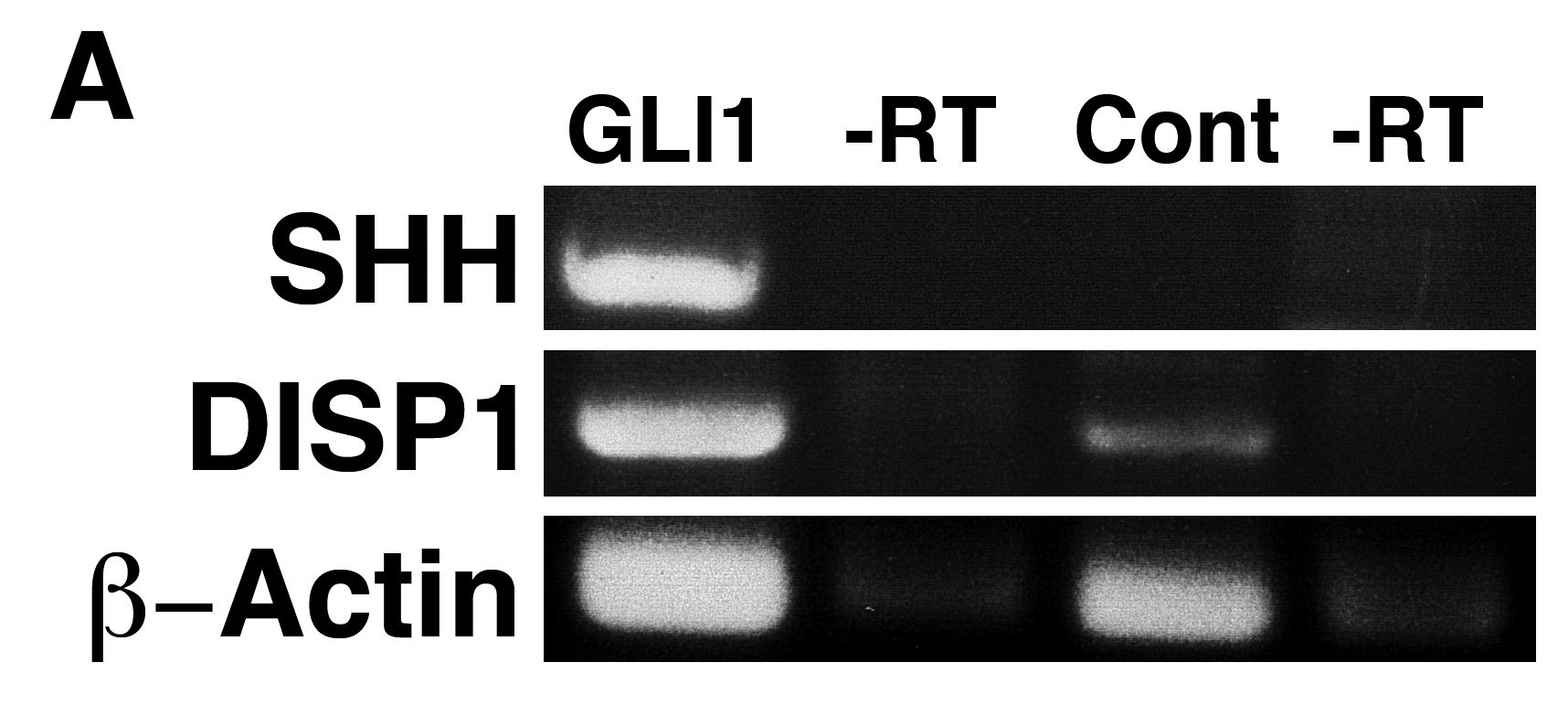

Supplement: Supplementary file 4 [file stem0028-1805-SD4.tif]

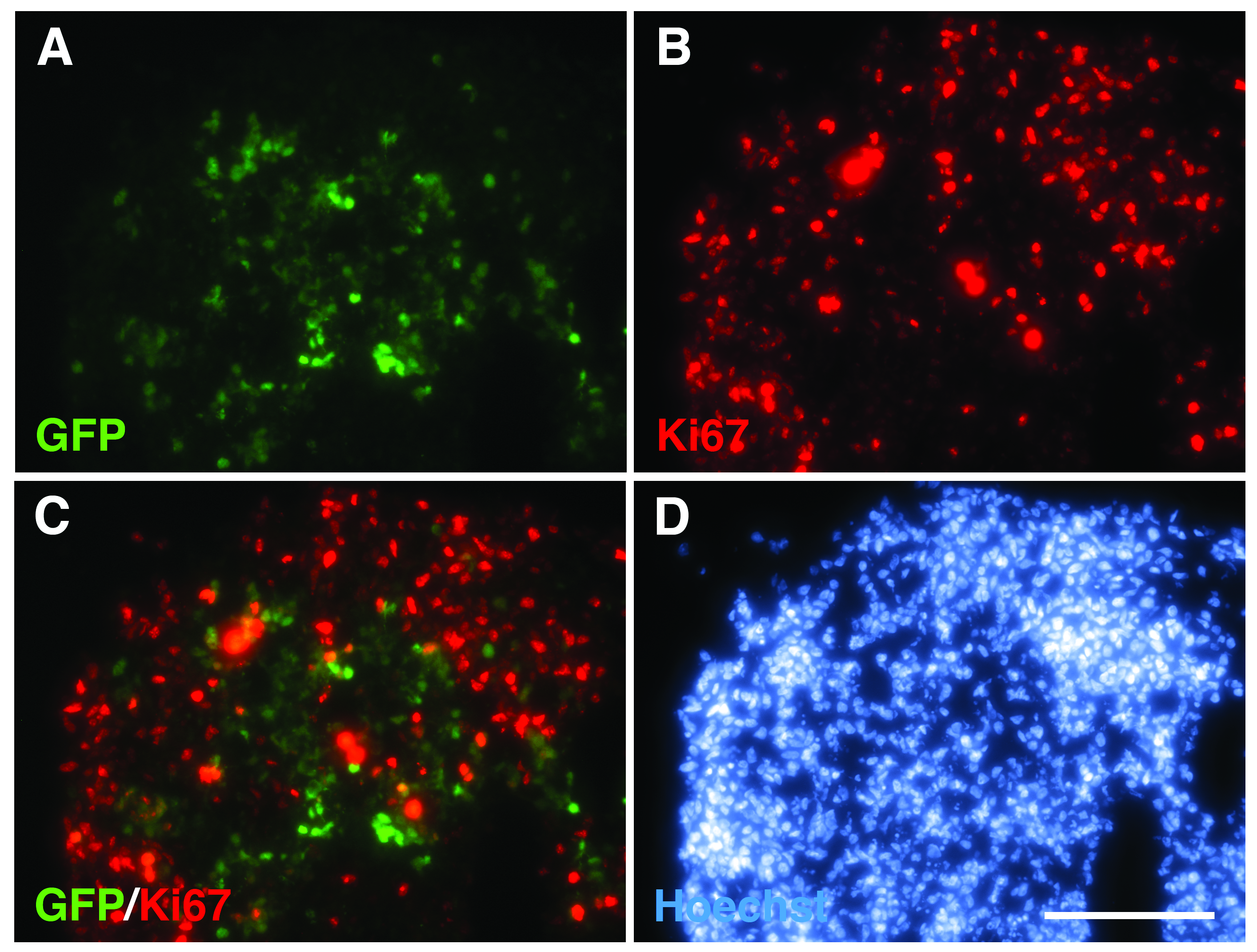

Supplement: Supplementary file 5 [file stem0028-1805-SD5.tif]

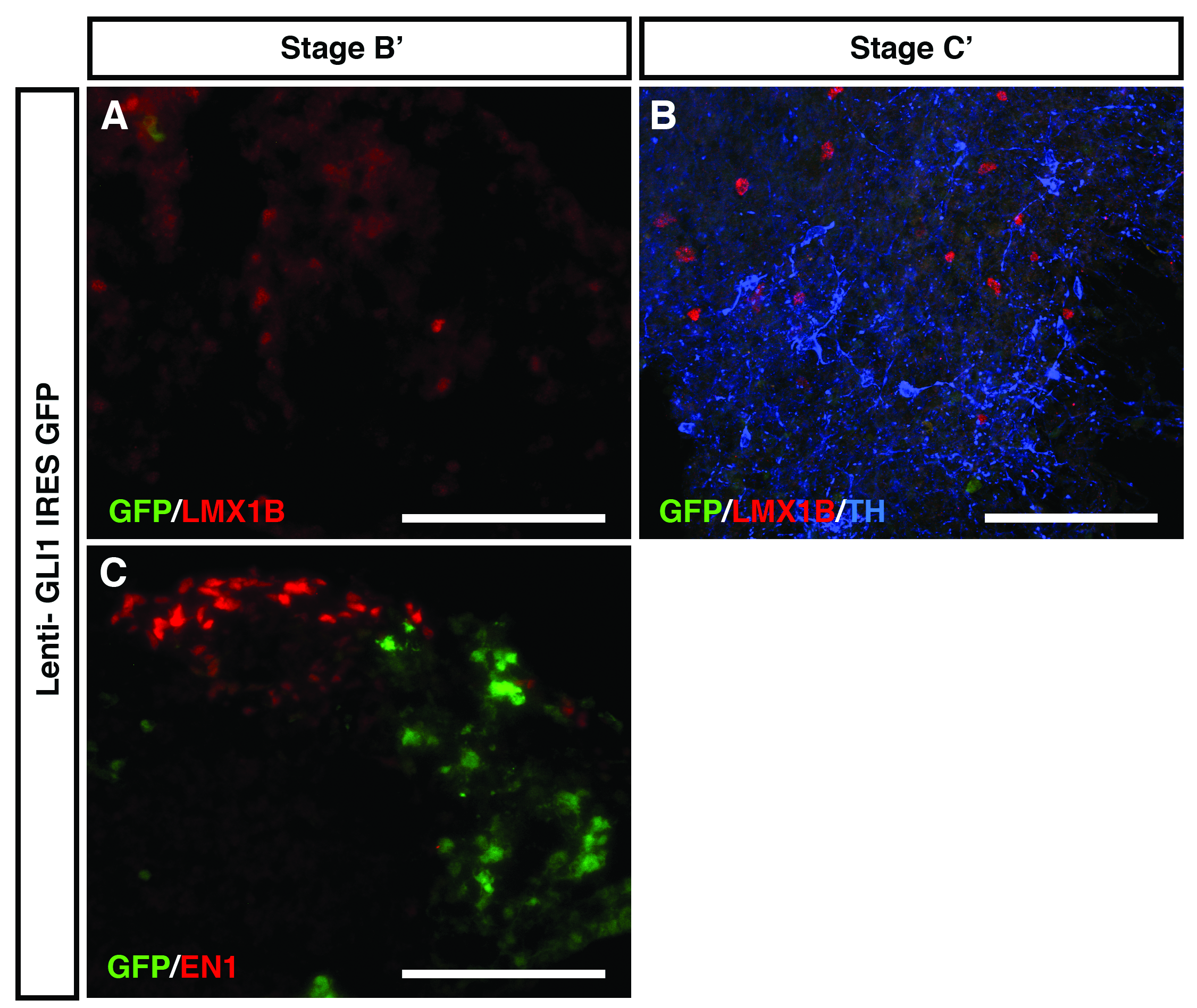

Supplement: Supplementary file 6 [file stem0028-1805-SD6.tif]
