# Supplementary material for: Gli1 Is an Inducing Factor in Generating Floor Plate Progenitor Cells from Human Embryonic Stem Cells
Source: Stem Cells. 2010 Aug 26;28(10):1805–15. doi: 10.1002/stem.510 (PMC2996857; doi:10.1002/stem.510)
Supplement: Supplementary file 7 [file stem0028-1805-SD7.doc]

**Supplementary Material and Methods**

**Reverse Transcription- Polymerase Chain Reaction (RT-PCR)**

Shh 618bp

Sense: AAAAGCTGACCCCTTTAGCC

Anti-sense: CCAGGAAAGTGAGGAAGTCG

Disp1 584bp

sense: CTGATCCATTGCTGGGTTTT

Anti-sense: GCACTTGAGCTGGTCCTTTC

Human beta-Actin 242bp

Sense: CACCACACCTTCTACAATGAGC

Anti-sense: TCGTAGATGGGCACAGTGTGGG

**Lentivirus Plasmid construction**

2K7-Neo plasmid was a kind gift from D. Suter and contains a R4-R2 gateway recombination cassette. The PGK-GFP fragment from the pRRL.sin-18.ppt.PGK.GFP vector was removed and the R4-R3 gateway cassette from pDest R4-R3 (Invitrogen) was inserted, generating the plasmid pRRL-3way. The following donor and entry vectors were recombined in various combinations with the above two lentiviral plasmids: pDonrP4-P1R-EF1α, pDonr221 EGFP, pEntry4 GLI1-IRES2GFP, pEntry4 GLI1, pDonrP2RP3-IRES-EGFP. Gateway recombination was performed according to standard protocols (Invitrogen). The final Lentiviral plasmids gnereated were as follows: 2K7 EF1α-EGFP Neo, 2K7 EF1α-GLI1-IRES-EGFP Neo, RRL EF1α-GLI1-IRES-EGFP.

**Supplementary Figure Legends**

**Figure S1.** **Comparison of PA6 neural induction system with GLI1 infected cells differentiated in defined media.** **(A-C)** TUJ1 FACS analysis of neurons. **(A)** PA6 induction system resulted in 92.37% (±1.38 SEM) of cells immunoreactive for TUJ1 at stage C. **(B)** GLI1 infected cells differentiated in defined media resulted in 80.91% (±1.60 SEM) of cells immunoreactive for TUJ1 **(C)** Isotype control for TUJ1 FACS analysis. **(D-F)** TH and FOXA2 FACS analysis of neurons. **(D)** PA6 induction system resulted in 8.07% (±0.78 SEM) TH+ and 0.40% (±0.29 SEM) TH+/FOXA2+. **(E)** GLI1 infected cells differentiated in defined media resulted in 12.09% (±4.82 SEM) TH+ and 1.63% (±0.24 SEM) TH+/FOXA2+

**Figure S2. Stage A’ neural differentiation of ENVY hES cells with 200ng/ml and 1000ng/ml of SHH-N added.** **(A-D)** The constitutively expressing GFP expressing hES cells line ENVY was differentiated to Stage A’ with the addition of SHH-N 200ng (A-B) and 1000 ng/ml (C-D) Very few FOXA2 positive cells are detected in either conditions (B-D). Scale bars: 100μm.

**Figure S3. StageA’ GLI1 infected ENVY cells were co-cultured with HES3 StageA’ neural cells for 12 days**. **(A-D)** FOXA2+/GFP- cells (arrow) are detected in close proximity to the GLI1 infected ENVY cells. Scale bars: 100μm.

**Figure S4.** **RT-PCR analysis of Gli1 and GFP control infected cells cultured to Stage B’.** **(A)** SHH transcripts were only detected in the GLI1 infected neurospheres. DISP1, a gene critically required for the secretion of SHH, was detected in both the GLI1 and control GFP infected cultures.

**Figure S5.** **Stage B’ GLI1 infected neurospheres were assessed for proliferating cells.** **(A-D)** Ki67-/GFP+ cells are detected within the neurospheres. Scale bars: 100μm.

**Figure S6. Expression analyses of mes DA markers in neurospheres and neurons derived from GLI1 transduced cultures. (A, C)** Few LMX1B+ (A) and Engrailed1+ (C) were detected in neurospheres (Stage B’), and none of these were GFP+. **(B)** Few LMX1B+ cells were detected in neurons (Stage C’). None of the LMX1B+ cells were also GFP+ or TH+. Scale bars: 100μm.
